# Poly(2-isopropenyl-2-oxazoline) as a Versatile Functional Polymer for Biomedical Applications

**DOI:** 10.3390/polym16121708

**Published:** 2024-06-14

**Authors:** Juraj Kronek, Alžbeta Minarčíková, Zuzana Kroneková, Monika Majerčíková, Paul Strasser, Ian Teasdale

**Affiliations:** 1 Department for Biomaterials Research, Polymer Institute of the Slovak Academy of Sciences, Dúbravská cesta 9, 845 41 Bratislava, Slovakia; alzbeta.minarcikova@savba.sk (A.M.); zuzana.kronekova@savba.sk (Z.K.); upolmoma@savba.sk (M.M.); 2 Institute of Polymer Chemistry, Johannes Kepler University, Altenbergerstrasse 69, 4040 Linz, Austria; paul.strasser@jku.at (P.S.); ian.teasdale@jku.at (I.T.)

**Keywords:** functional polymers, poly(2-isopropenyl-2-oxazoline), drug delivery, gene delivery, orthogonal chemistry

## Abstract

Functional polymers play an important role in various biomedical applications. From many choices, poly(2-isopropenyl-2-oxazoline) (PIPOx) represents a promising reactive polymer with great potential in various biomedical applications. PIPOx, with pendant reactive 2-oxazoline groups, can be readily prepared in a controllable manner via several controlled/living polymerization methods, such as living anionic polymerization, atom transfer radical polymerization (ATRP), reversible addition–fragmentation transfer (RAFT) or rare earth metal-mediated group transfer polymerization. The reactivity of pendant 2-oxazoline allows selective reactions with thiol and carboxylic group-containing compounds without the presence of any catalyst. Moreover, PIPOx has been demonstrated to be a non-cytotoxic polymer with immunomodulative properties. Post-polymerization functionalization of PIPOx has been used for the preparation of thermosensitive or cationic polymers, drug conjugates, hydrogels, brush-like materials, and polymer coatings available for drug and gene delivery, tissue engineering, blood-like materials, antimicrobial materials, and many others. This mini-review covers new achievements in PIPOx synthesis, reactivity, and use in biomedical applications.

## 1. Introduction

**Figure 1:**
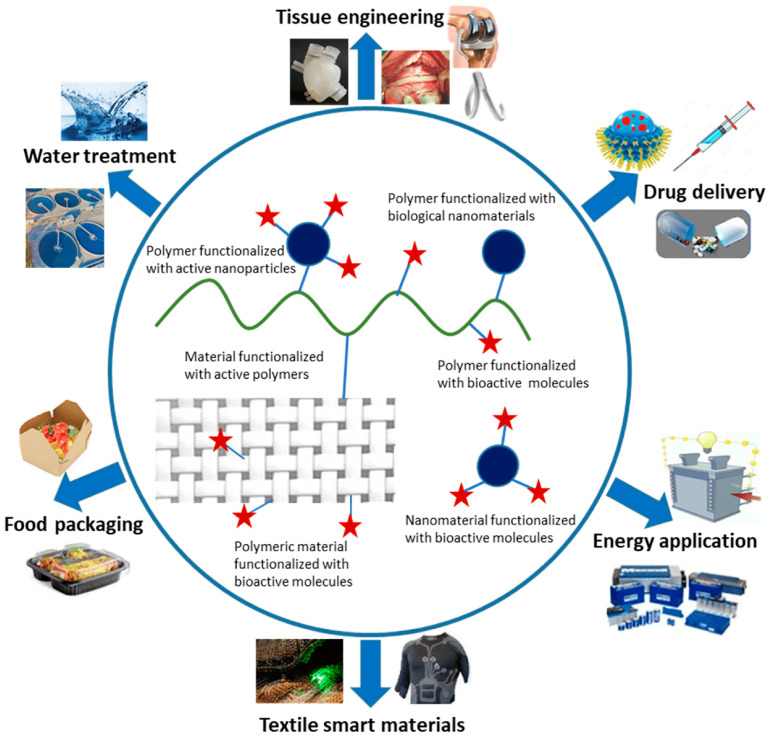
Application possibilities employing functional polymeric materials. Reprinted from Ref. [1].

Recently, functional polymers have gained an important position in various technological applications, for example, wastewater treatment, food packaging, cosmetics, smart textiles, and energy storage, as well as many biomedical applications, including drug and gene delivery, tissue engineering, and non-biofouling protective coatings (Figure 1) [1]. Functionalized polymeric materials are widely studied in many biomedical applications mainly due to their diverse technical characteristics and adaptable properties. For example, many functional polymeric materials have been developed to improve the performance of medical diagnostics through various approaches, including the enhancement of contrast in imaging technologies and the promotion of molecular recognition in advanced diagnostic assessments. Functional polymers have also gained an important role in personalized medicine, drug discovery, slow-release therapeutics, immunoprotection, and immunostimulation agents [2,3].

Each biomaterial intended for in vivo performance has to fulfill specific requirements. To date, a plethora of different polymer structures with different chemistry and functionalities are available on the market. From the biopolymers, different polysaccharides, such as chitosan, hyaluronic acid, or sodium alginate, have been used as functional polymers for drug and gene delivery, regenerative medicine, or tissue engineering [4,5,6,7]. On the other hand, many synthetic polymers with different functionalities are considered promising biomedical polymers. For example, substituted (meth)acrylamides, such as poly(2-hydroxypropylmethacrylamide) (PHPMA), represent functional synthetic polymers used as smart polymers or drug carriers [8,9,10]. PHPMA-doxorubicin conjugates were demonstrated as efficient anticancer therapeutic agents [11]. Poly(ethylene glycol) is widely used for the covalent attachment of polymer chains to bioactive substances, such as proteins or drugs, in the process known as PEGylation [12,13]. Other examples of functional polymers include poly(N-vinylpyrrolidone) (PVP) [14] or substituted poly(phosphazenes), a unique class of hybrid inorganic–organic polymers, with a range of applications including tissue engineering and drug delivery, such as hydrogels, shape memory polymers, stimuli, and responsive materials [15,16].

**Figure 2:**
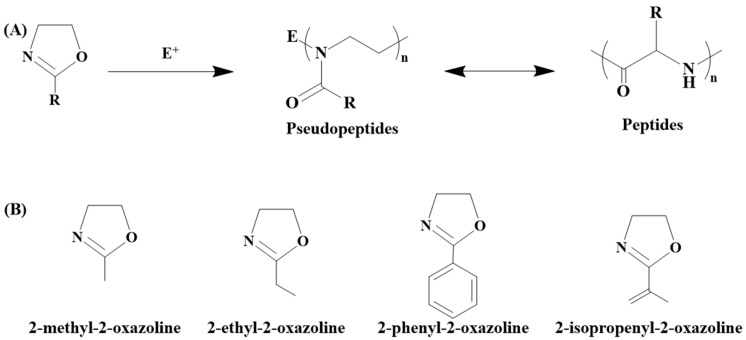
Scheme of cationic polymerization of 2-oxazolines (**A**) and structures of commercially available 2-oxazoline monomers (**B**).

In recent years, poly(2-oxazoline)s (POx) have gained prominence as a versatile and promising type of polymer as they exhibit unique properties and functionalities, making them suitable for various applications. POx belongs to the category of tertiary polyamides, with the amide group located in the side chain (Figure 2A).

This structural arrangement positions them as isomers of polypeptides, categorizing them as pseudopeptides [17,18]. POx is most often prepared by the method of living cationic ring-opening polymerization of 2-substituted-2-oxazolines, which allows for the controlled synthesis of POx with specific structures, functionalities, and adjustable physicochemical properties. The nature of the resultant poly(2-oxazoline)s can be regulated by changing the 2-substituent of the 2-oxazoline monomer, which serves as the polymer’s side chain. This flexibility in the side chain affords the ability to attain a broad spectrum of polymer properties, encompassing hydrophilic, hydrophobic, and fluorophilic characteristics. Moreover, it facilitates the development of polymers with varying degrees of flexibility, ranging from highly flexible to semi-crystalline or relatively rigid, and creates responsive polymers. Beyond the preparation of living cationic ring-opening polymerization, POx can also be synthesized using other polymerization techniques [18,19,20,21]. Generally, a variety of 2-oxazoline monomers has become commercially accessible, including compounds such as 2-methyl-2-oxazoline (MeOx), 2-ethyl-2-oxazoline (EtOx), 2-phenyl-2-oxazoline, or 2-isopropenyl-2-oxazoline (IPOx) (Figure 2B). Although the 2-oxazoline ring-containing compound was synthesized for the first time in 1884 by Andreash [22], this type of compound started to have a real utilization in polymer chemistry in the 1960s when Litt, Kagiya, Tomalia, and Seeliger published the successful cationic polymerization of 2-methyl-2-oxazoline [23,24,25,26], as surveyed in Table 1. 2-isopropenyl-2-oxazoline (IPOx) was first polymerized by free-radical polymerization in 1972 [27].

From a variety of polymers based on 2-oxazoline chemistry, poly(2-isopropenyl-2-oxazoline) (PIPOx), prepared by vinyl polymerizations, represents an interesting class of functional polymers with excellent biocompatibility and immunomodulatory properties. Here, anionic polymerization (AP), free-radical polymerization, atom transfer radical polymerization (ATRP), reversible addition–fragmentation chain transfer polymerization (RAFT), copolymerization, etc., have been explored to create POx with distinct characteristics. These alternative approaches offer additional avenues for tailoring the properties of POx to meet specific requirements.

**Figure 3:**
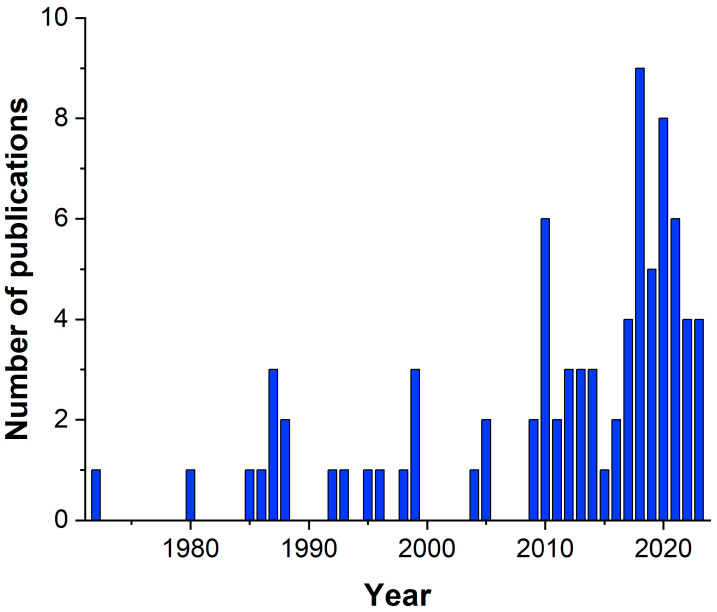
The number of publications containing the keyword “2-isopropenyl-2-oxazoline” collected from the Scopus database. Available online: https://www.scopus.com/term/analyzer.uri?sort=plf-f&src=s&sid=ccd6e54a6e9ea3263e0d454c409ffbb8&sot=a&sdt=a&sl=40&s=TITLE-ABS-KEY%282-isopropenyl-2-oxazoline%29&origin=resultslist&count=10&analyzeResults=Analyze+results (accessed on 20 May 2024).

In 1972, Kagiya described the preparation of hydrogels by the addition reaction of PIPOx with adipic acid [28]. In 1980, Tomalia demonstrated the anionic polymerization of IPOx as a way to prepare polymers with pendant 2-oxazoline rings [29]. Surprisingly, the first practical application of PIPOx was published thirteen years later by Baker, who used PIPOx for the compatibilization of polymer blends [30]. The tremendous potential of PIPOx in biomedical applications was unraveled in 2016 when we demonstrated PIPOx as an in vitro non-cytotoxic polymer [31]. From that time, several studies reported examples of the possible applications of PIPOx in various biomedical fields, like drug and gene delivery, hydrogel technologies, antimicrobial surfaces, and others. Moreover, from the publication activities displayed in Figure 3, we can observe continuously increasing interest in PIPOx research in the last decade. This trend declares the great importance of poly(2-isopropenyl-2-oxazoline) in various fields of polymer chemistry.

Therefore, this mini-review surveys information not only on the synthesis and reactivity of PIPOx but also on the application potential of polymers based on 2-isopropenyl-2-oxazoline.

**Table 1:** Historical milestones in polymer chemistry of 2-isopropropenyl-2-oxazoline (IPOx) and applications of poly(2-isopropenyl-2-oxaozline) as promising biomaterials.

Year	Milestone	Authors	Ref.
1884	First synthesis of 2-oxazoline cycle from allylurea	Andreash	[22]
1966	Cationic polymerization of 2-methyl-2-oxazoline	Kagiya, Litt, Tomalia, Seeliger	[23,24,25,26]
1972	Free-radical polymerization of IPOx	Kagyia	[27]
	Hydrogels based on PIPOx	Kagiya	[28]
	Addition reactions of PIPOx with carboxylic acids	Kagiya	[28]
1980	Anionic polymerization of IPOx	Tomalia	[29]
1993	Compatibilization of polymer blends using PIPOx	Baker	[30]
1996	Selective addition reactions of PIPOx with thiols and carboxylic acids	Nishikubo	[32]
2009	Polymer brushes based on PIPOx	Jordan	[33]
2010	Controlled radical polymerization of IPOx	Schubert	[34]
2013	Group-transfer polymerization of IPOx	Rieger	[35]
2016	In vitro cytotoxicity studies of PIPOx	Kronek	[31]
2018	Plasma-polymerized PIPOx coatings	Zanini	[36]
	Thermosensitive polymers based on PIPOx	Hoogenboom	[37]
2020	ATRP of PIPOx	Raus	[38]
	Drug delivery systems based on PIPOx	Hoogenboom	[39]
2021	Segmented networks based on PIPOx	Basko	[40]
	In vitro hydrolytic stability of PIPOx	Hoogenboom	[41]
2023	Hybrid metal nanoparticles based on PIPOx	Mosnáček	[42]
	PIPOx polymers for gene delivery	Hoogenboom	[43]

## 2. Synthesis of 2-Isopropenyl-2-Oxazoline

**Figure 4:**
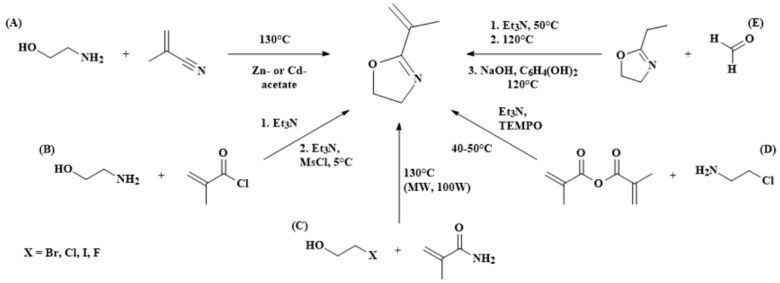
Schematic summary of selected synthetic pathways towards the 2-isopropenyl-2-oxazoline (IPOx) monomer. (**A**) Synthesis based on 2-aminoethanol and methacrylonitrile; (**B**) Synthesis based on 2-aminoethanol and methacryloyl chloride; (**C**) Synthesis based on a haloalkanol and methacrylamide; (**D**) Synthesis based on 2-chloroethylamine and methacrylic anhydride; (**E**) Synthesis based on 2-ethyl-2-oxazoline and formaldehyde.

The monomer 2-isopropenyl-2-oxazoline (IPOx) is commercially available and typically purified before use by drying over CaH_2_ or 2,6-di-tert-butyl-4-methylphenol, for example, followed by vacuum distillation [44,45]. Furthermore, several different synthetic procedures for IPOx are reported in the literature, as summarized in Figure 4 [46]. Research on the synthesis of 2-oxazolines has been performed for many decades, with extensive reports detailing the preparation of various oxazoline derivatives along with their different reactions and applications [47,48,49]. Hoogenboom and Schubert et al. reported the screening of twenty-nine 2-oxazoline derivatives based on the condensation reaction of nitriles with 2-aminoethanol and zinc acetate or cadmium acetate as the catalyst (Figure 4A) [50]. Although IPOx and methacrylonitrile have a boiling point below the reaction temperature of 130 °C, leading to some loss of substance, product formation was observed, indicating the general suitability of the synthetic approach. Nevertheless, in addition to this screening experiment, IPOx synthesis from methacrylonitrile was not investigated in detail. Methacryloyl chloride, a significantly cheaper alternative to methacrylonitrile, can also be reacted with 2-aminoethanol in a two-step reaction (Figure 4B) [51]. After initial amide formation at room temperature, ring closure is subsequently performed with methanesulfonyl chloride at a temperature of 5 °C. The crude product is purified by simple aqueous washing and a short-path vacuum distillation. However, only a low yield of 17% could be achieved.

Several patents describing the synthesis of 2-oxazolines have been filed as well. For instance, methacrylamide can be reacted with a haloalkanol, either microwave assisted or at elevated temperatures of around 130 °C, yielding the desired 2-isopropenyl-2-oxazoline, as seen in Figure 4C [52]. Along with low-cost reagents, the microwave-assisted procedure provides significantly reduced reaction times of only 40 min with one of the highest reported yields of around 75%. Alternatively, 2-chloroethylamine reacts with methacrylic anhydride at around 40–50 °C in the presence of Et_3_N, and (2,2,6,6-tetramethylpiperidin-1-yl)oxyl is used as a polymerization inhibitor (Figure 4D) [53]. While the reaction duration is prolonged to around 18h, a lower reaction temperature can be used. The monomer can be isolated by vacuum distillation with a yield of around 60% after a simple filtration. Finally, the IPOx monomer can also be received by derivatization of the simpler 2-ethyl-2-oxazoline (EtOx). To this end, EtOx is treated with formaldehyde in the presence of Et_3_N under reflux, reacts with NaOH and hydroquinone at 120 °C, and is isolated by vacuum distillation (Figure 4E) [54]. Although the general structural framework with EtOx was provided, only a yield of 36% could be achieved. In addition, the necessity of an intermediate purification step by vacuum distillation further complicates this path. Overall, apart from providing the highest yields of around 75% and 60%, the proposed methods employing methacrylamide and methacrylic anhydride (Figure 4C,D), respectively, appear to offer the most straightforward synthetic approach towards 2-isopropenyl-2-oxazoline.

## 3. Polymerization Methods of 2-Isopropenyl-2-Oxazoline

**Figure 5:**
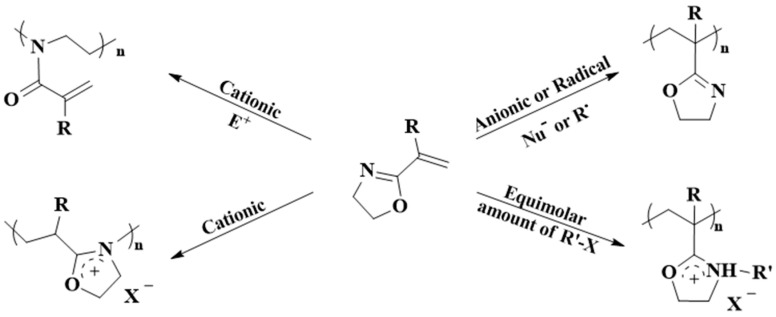
Schematic summary of different synthetic pathways for the polymerization of 2-isopropenyl-2-oxazoline, depending on the reaction conditions.

IPOx represents a bifunctional monomer with two orthogonal functionalities, and polymerization may occur on the 2-oxazoline unit or the double bond. The monomer can be polymerized depending on reaction conditions by living anionic polymerization, cationic ring-opening polymerization, radical polymerization, RAFT, or spontaneous polymerization, resulting in polymers with different chemical structures (Figure 5) [55].

It is known that the cationic polymerization of different 2-oxazoline monomers performed via a living mechanism forms poly(N-acylethylene imines) [56]. Living cationic ring-opening polymerizations (LCROPs) are typically performed in polar aprotic solvents, such as acetonitrile, benzonitrile, sulfolane, or N,N-dimethylacetamide, using different electrophiles as initiators. From a broad group of possible cationic initiators, alkyl halogenides and alkyl sulfonates are the most widely used [57]. Cationic polymerization of IPOx initiated by HClO_4_ is very sensitive to the presence of free radicals [28]. The linear polymer was only obtained in the case when polymerization was carried out in the presence of hydroquinone at temperatures ranging from 70 to 125 °C. On the other hand, Saegusa et al. reported an interesting ring-preserved polymerization using N-protio-2-isopropenyl-2-oxazolinium salt, which has a close similarity to N-methyl salt [58].

**Figure 6:**
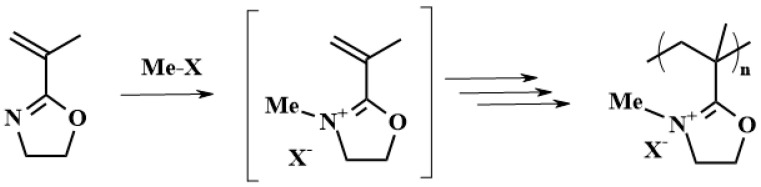
Scheme of vinyl polymerization of 2-isopropenyl-2-oxazoline in the presence of oxazolinium salts.

Vinyl polymerization of IPOx has been performed in the presence of Lewis acids [27] and of 2-oxazolinium salts [59]. In contrast to the cationic polymerization of IPOx, vinyl polymerization proceeded at low temperatures and in less polar solvents (Figure 6). Poly(2–isopropenyl–2–oxazoline) (PIPOx) with pendant 2–oxazoline rings in the side chain has traditionally been prepared by free-radical polymerization initiated by AIBN, resulting in polymers with broad dispersity and low control over molar masses. The polymerization is typically performed in N,N-dimethylacetamide at 60 °C for 8 h with conversions up to 60% and a dispersity ranging from 1.8 to 2.0 [31,38,60,61].

Higher-dispersity PIPOx was also achieved in the polymerization of IPOx by Al-based Frustrated Lewis pairs [62]. All polymerizations were carried out at ambient temperature (ca. 25 °C) in toluene, achieving PIPOx in high conversions ranging from 80 to 99% and dispersities ranging from 2.9 to 3.3. Better results were achieved when 2-vinylpyridine was used as a monomer.

Rieger and colleagues reported the rare earth metal-mediated groups-transfer polymerization of IPOx, yielding polymers and copolymers of various architecture [35,63]. In this manner, PIPOx has been efficiently synthesized using bis(cyclopentadienyl)methylytterbium as a catalyst. The polymerizations of 2-isopropenyl-2-oxazoline (IPOx) followed a living group-transfer polymerization mechanism, allowing precise control over molar masses with very narrow dispersities. Well-defined PIPOx has also been prepared by living anionic polymerization initiated by n–butyllithium [22] or a diphenylmethylpotassium/diethylzinc initiation system [64].

**Figure 7:**
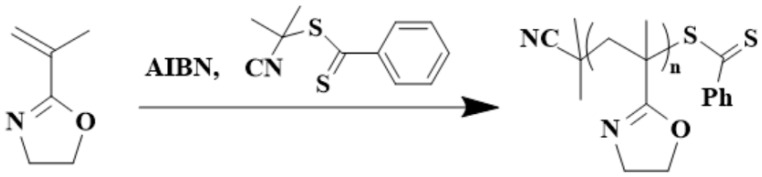
The scheme of reversible addition–fragmentation transfer polymerization of 2-isopropenyl-2-oxazoline initiated by AIBN using dithiobenzoate as a transfer agent.

Reversible addition–fragmentation transfer (RAFT) polymerization of IPOx was reported as another type of controlled polymerization method (Figure 7) [34]. Here, three different types of chain transfer agents (dithiocarbamate, dithiobenzoate, and tritiocarbonate) have been used. The reaction proceeded in a 2 M toluene solution at 70 °C for 18 h using AIBN as an initiator. The best results were obtained for the system comprising dithiobenzoate, where PIPOx with a dispersity of 1.38 was achieved. The polymerization was significantly slowed down at 30% monomer conversions. This may be due to the formation of an overly stable intermediate layer after initiation of AIBN and thus the inability to re-initiate polymerization.

The first attempts to polymerize iPOx via atom transfer radical polymerization (ATRP) were unsuccessful [33]. The authors stated that the limited conversion and formation of oligomers were caused by strong interactions of copper with 2–oxazoline rings. Contrary to this statement, Raus and colleagues reported successful aqueous copper(0)-mediated reversible deactivation radical polymerization (Cu^0^–RDPR) of IPOx with a polymerization mechanism related to ATRP [38]. Polymerizations were performed in a controlled way using the 2–chloropropionitrile/CuCl/CuCl_2_/TPMA initiation and catalytic system, and polymerizations were carried out in 0.67 M aqueous NaCl. The authors demonstrated that polymerization is highly sensitive to the initiator concentration and the CuCl/CuCl_2_ ratio. On the other hand, careful optimization of the polymerization parameters can lead to narrow-dispersity polymers, maintaining almost quantitative conversions. Depending on polymerization conditions, dispersities of the resulting polymers were achieved with values close to 1.10. Similarly, ATRP was also performed in polar organic solvents, such as DMSO, DMAc, DMF, or anisole [42]. Polymerizations proceeded in high conversions and polymers with controlled molar masses up to 20,000 g/mol, and dispersities in the range of 1.2–1.5 have been achieved. In this polymerization set up, the 2–chloropropionitrile/CuCl/CuCl_2_/TPMA initiation and catalytic system was used. The surface-initiated atom transfer radical polymerization (SI-ATRP) of iPOx under the same polymerization conditions was used for the surface modification of magnetic carbonyl iron–core–shell particles with two different molar masses of the grafted PIPOx. Surface-initiated reversible deactivation radical polymerization (SI-RDRP) of IPOx has also been used for the preparation of bottle-brush brushes [65].

## 4. Post-Polymerization Modification of Poly(2-isopropenyl-2-oxazoline)

**Figure 8:**
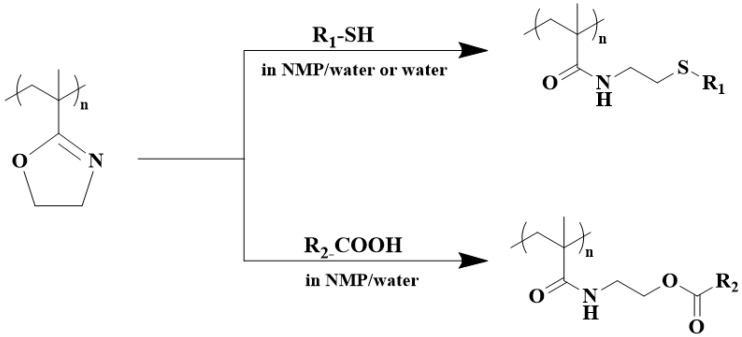
Schematic presentation of possible addition reactions of PIPOx with different carboxylic acids and thiols.

PIPOx with pendant 2-oxazoline groups has been recognized as a universal platform for the preparation of functional polymeric materials for various applications by the ring-opening addition reaction of the 2-oxazoline ring with carboxylic acids at elevated temperatures, leading to derivatives with an amide–ester linkage. For example, the equimolar mixture of PIPOx and butyric acid at 150 °C in N,N-dimethylacetamide gave a white powdery solid of a polymer with a secondary amide structure, as confirmed by FTIR spectroscopy [28]. Nishikubo and colleagues demonstrated a selective addition reaction of 2-oxazoline units with selected thiols and carboxylic groups [32]. They showed that addition reactions of 2-oxazoline rings with aromatic and aliphatic carboxylic acids and thiols were possible in polar aprotic solvents and also in aprotic solvent/water mixtures. Moreover, the addition reaction of PIPOx with thiols even works in aqueous solutions (Figure 8).

Recently, addition reactions with carboxy groups have been used not only for organic molecules like drugs, fluorescent dyes, or light-sensitive moieties but also for coupling reactions with polymers containing carboxy units. Possible post-polymerization modifications and used reaction conditions were surveyed in a mini-review written by Basko and colleagues [46].

## 5. Poly(2-isopropenyl-2-oxazoline) in Biomedical Applications

### 5.1. Biocompatibility and Immunocompatibility of Poly(2-isopropenyl-2-oxazoline)

As already mentioned, poly(2-isopropenyl-2-oxazoline) prepared by vinyl polymerizations contains pendant 2-oxazoline units, suitable for further functionalization. This feature makes them an ideal polymer platform in drug and gene delivery, tissue engineering, hydrogel technology, or smart polymeric materials [46]. Moreover, PIPOx was verified as a biocompatible and immunomodulative polymer in several independent studies [31,45,66,67]. PIPOx prepared by free-radical polymerization with an M_n_ of 21,000 g·mol^−1^ and Ɖ = 1.85 was shown to be non-cytotoxic up to a concentration of 10 mg·mL^–1^ [24]. For the in vitro cytotoxicity study determined by the MTT assay, murine 3T3 fibroblasts and P388D1 macrophage-like cells have been used. PIPOx with an M_n_ of 10,000 g·mol^–1^ prepared by anionic polymerization was non-cytotoxic, even up to 50 mg·mL^–1^ using L929 murine fibroblast cells [66]. PIPOx of different molecular weights prepared by atom transfer radical polymerization was also tested for its cytotoxicity on 3T3 fibroblasts, as well as on 3D reconstructed human tissue models of epidermal and epiintestinal tissues [67]. It was shown that PIPOx with a molar mass ranging from 3000 g·mol^−1^ to 45,000 g·mol^−1^ is not cytotoxic to both tissues up to the highest tested concentration of 10 mg·mL^−1^. Interestingly, PIPOx modified carbonyl-iron particles containing PIPOx with two different molar masses showed a high level of cytocompatibility, although particles modified with short PIPOx (M_n_ = 1470 g·mol^−1^) were more cytotoxic to 3T3 fibroblasts compared to particles containing PIPOx with M_n_ = 17,000 g·mol^−1^ [42]. Thus, the cytotoxicity of PIPOx depends on its molecular weight and increases with an M_n_ below 3000 g·mol^−1^. Significant differences in cell proliferation were observed for macrophages. It was shown in two independent studies that PIPOx stimulates macrophage cell proliferation in a free form [31], and also if grafted onto carbonyl iron nanoparticles [42]. Moreover, the induction of macrophage cell proliferation seems to be PIPOx size dependent; the higher the molecular weight of PIPOx, the more that cell proliferation was increased [42], suggesting that PIPOx has immunomodulatory properties.

Ex vivo studies were conducted on mice splenocytes to explain the immunomodulatory properties of PIPOx [31,45,56]. Different immunocompetent cells, such as CD11c^+^ and CD14^+^ cells, represented by monocytes, granulocytes, dendritic cells, and macrophages, as well as CD4^+^ cells, represented by non-adherent T cells, were stimulated with PIPOx. The polarization of the immune system was evaluated based on the expression of signature Th1 (IFN-γ), Th2 (IL-4), Th17 (IL-17), and Treg (IL-10) cytokines. The published results suggest that PIPOx can accelerate cell-specific immune responses. PIPOx-sensitized adherent CD11c+ and CD14+ spleen cells produced significantly elevated cytokines, IFN-γ and IL-17, indicating the polarization of immune cell response towards Th1/Th17 protective immunity, which is important for the proinflammatory responses, killing intracellular parasites, and maintaining autoimmune responses. On the other hand, adherent spleen cell-derived CD11c+-enriched antigen-producing cells (APCs) show a statistically significantly higher secretion of IL-10, the signature cytokine of the Treg phenotype that has the opposite anti-inflammatory response.

The non-inflammatory character of PIPOx and its drug conjugates was shown by Kronekova et al., 2024, studying tissue-specific immunity using 3D tissue models of epiderm (Epiderm^TM^, MatTek IVLSL, Bratislava, Slovakia) and intestinal epithelium (EpiIntestinal^TM^, MatTek) and evaluating the production of selected proinflammatory interleukins and growth factors, like TNFα, IL-6, IL-8, IL-1 α, and ß [67].

**Figure 9:**
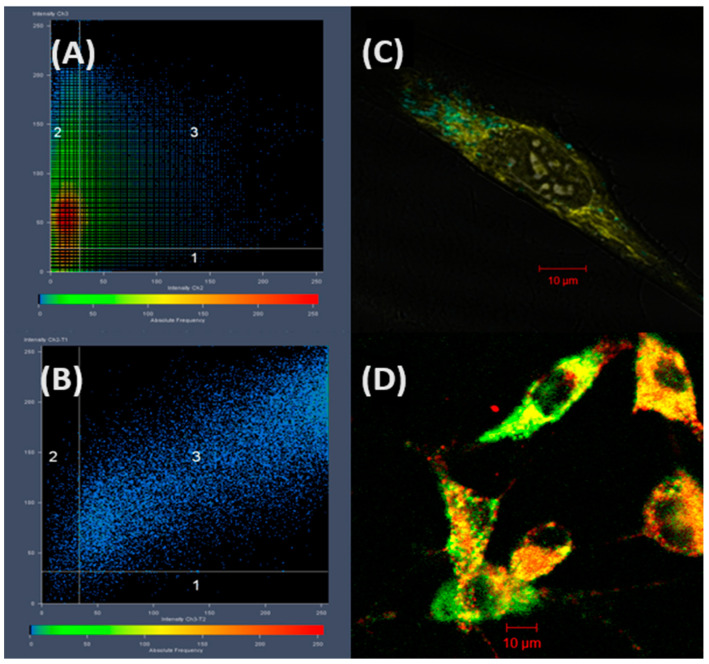
ROI analysis of PIPOx colocalization with (**A**) mitochondria and (**B**) lysosomes. (**C**) Merged images of fluorescently labeled PIPOx (cyan) and MitoTracker labeled mitochondria (yellow). (**D**) A merged image of fluorescently labeled PIPOx (green) and LysoTracker labeled lysosomes (red). Reprinted with permission from Ref. [31]. Copyright 2016 John Wiley and Sons.

Confocal laser scanning microscopy showed that PIPOx can internalize fibroblasts and macrophages. These studies confirmed that PIPOx is not freely distributed in the cytoplasm but is located in cell vesicular structures (Figure 9). The higher distribution of PIPOx within the lysosomes compared to other vesicular structures demonstrates that fluorescently labeled PIPOx remains present in the early or late endosomes, which are the first compartments in the endocytic pathway [31,45].

Jerca et al. also studied protein adsorption on PIPOx hydrogels. The ability to repel proteins within aqueous environments would be highly beneficial in preventing many undesired polymer–cell and polymer–microorganism interactions, known as anti-fouling behavior. The amount of protein adsorption from 0.5 mg/mL BSA and 1 mg/mL BSA solutions was very small, close to the detection limit for the Coomassie colloidal blue G250 staining [66].

For the future use of PIPOx-based polymeric materials, such as drug conjugates or hydrogels, their chemical stability in physiologically relevant media is also important. Jerca et al. systematically studied the hydrolytic stability of PIPOx as a function of pH in different buffers [41]. PIPOx was found to be stable for 2 to 3 weeks in basic conditions, 5 days in neutral conditions, and hours in acidic conditions before the beginning of the degradation process.

### 5.2. Thermosensitive Polymers

Thermosensitive polymers can respond to temperature changes by changes in solubility and are the most studied type of smart materials [68]. The majority of the examples involve thermoresponsive polymeric systems exhibiting a lower critical solution temperature (LCST) [69]. In this regard, the N-substituted polyacrylamides [70], poly(oligoethyleneoxide (meth)acrylate)s [71], and poly(2-alkyl-2-oxazoline)s [72] are the most pronounced thermosensitive polymers. These polymers can also exhibit a response in the physiologically relevant window, which is a desirable feature for various biomedical applications, such as drug delivery, tissue engineering, regenerative medicine, and biosensing [73].

**Figure 10:**
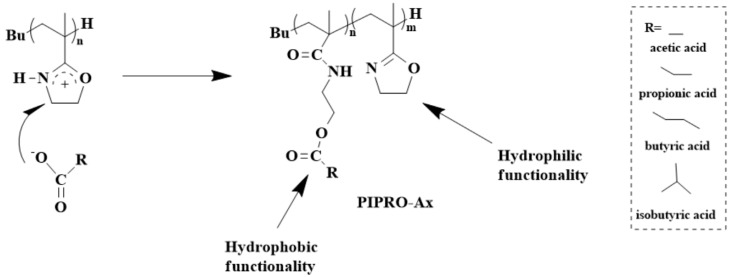
Synthesis of the thermosensitive polymers via the addition reaction of PIPOx with various carboxylic acids.

**Figure 11:**
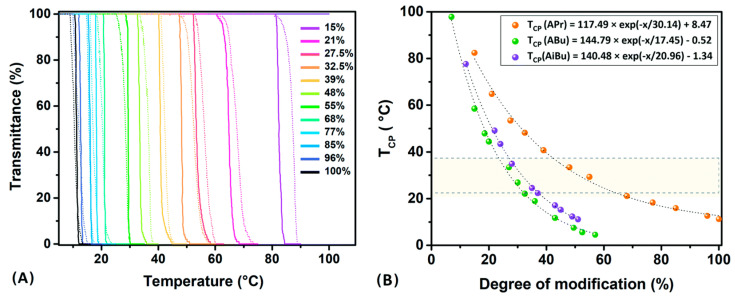
Transmittance versus temperature profiles for the second heating (solid lines) and cooling (dashed lines) cycles of PiPOx-APr series in distilled water (10 mg/mL; 0.5 °C/min) (**A**). The effect of the acid content on the cloud point temperatures (TCP) for the PiPOx acid series (**B**). Reprinted with permission from Ref. [37]. Copyright 2010 Royal Society of Chemistry.

PIPOx represents a highly hydrophilic polymer soluble in water and relevant physiological media in a large temperature range. Hoogenboom et al. demonstrated that the hydrophobic modification of PIPOx can lead to the preparation of thermosensitive polymers with LCSTs ranging from 5 to 97 °C. In this work, PIPOx was modified with four different carboxylic acids, namely, acetic acid, propionic acid, butyric acid, and isobutyric acid. (Figure 10) [37]. The modulation of the cloud point temperature (TCP) has been achieved by controlling the modification degree and by changing the hydrophobic character of the acid (Figure 11).

### 5.3. Drug Conjugates

Drug-delivery systems present an immense interest in the research of functional biomedical polymers [74,75,76]. They can be principally divided into physically entrapped drugs in hydrogels or nanoparticles and covalently bound drugs in drug conjugates. On the one hand, drug conjugates can be prepared by the reaction of end groups, as reported for drug PEG conjugates [77]. Side chain functionalization serves as a possibility to prepare drug conjugates with a higher concentration of a given drug. In such a manner, conjugates of poly(2-hydroxypropylacrylamide) (PHPMA) with doxorubicin have been prepared and used for highly selective treatment of cancer diseases [8,9,78].

PIPOx, containing pendant 2-oxazoline rings, can provide an additional reaction to drugs containing carboxylic groups, such as ibuprofen or aspirin. Kronekova et al. prepared conjugates of PIPOX, prepared by copper (0)-mediated reversible deactivation radical polymerization with four different molar masses ranging from 3000 to 45,000 g·mol^−1^, with ibuprofen in N,N-dimethylacetamide at 150 °C for 5 h [67]. Ibuprofen-PIPOx conjugates up to 5 mol% were fully soluble in water and physiological fluids, and no aggregation was observed in dynamic light scattering measurements.

**Figure 12:**
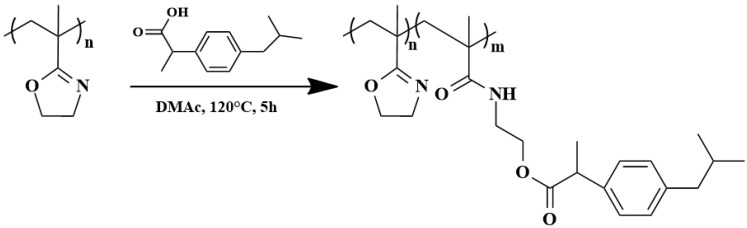
Preparation of ibuprofen PIPOx conjugates through the addition reaction of PIPOx with ibuprofen.

**Figure 13:**
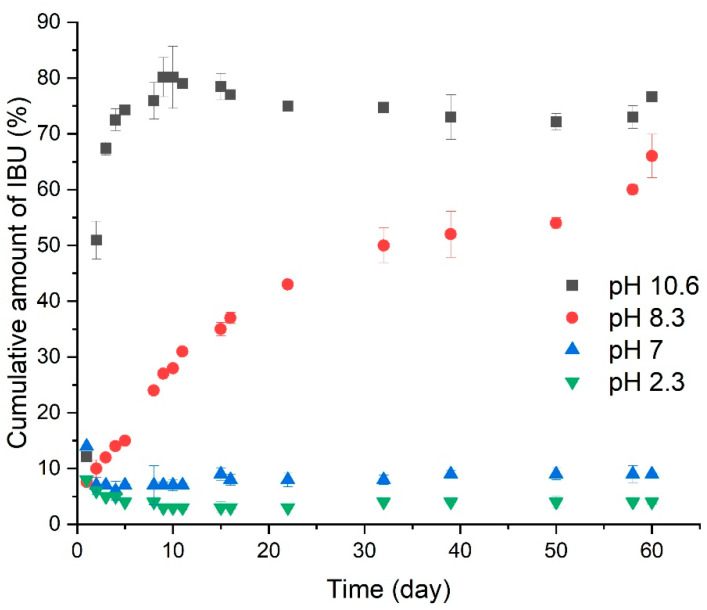
Release profiles of ibuprofen from the polymer drug conjugate in vitro at 37 °C, as determined by HPLC. Adapted from Ref. [67].

The addition of ibuprofen to the 2-oxazoline ring led to the formation of hydrolyzable ester–amide linkages (Figure 12). The release of ibuprofen in buffers with different pH was monitored using C18 reverse-phase high-performance liquid chromatography (RP HPLC). The highest rate of ibuprofen release was observed at pH = 10.6 while at low pH, no release was observed (Figure 13). In vitro studies on reconstructed EpiDerm and EpiIntestinal 3D tissue models demonstrated no harmful effects of ibuprofen PIPOx conjugates on the viability and morphology of both model tissues compared to free ibuprofen.

### 5.4. Cationic Polymers

Cationic polymers have received an important role in various applications, such as drug and gene delivery systems, antimicrobial agents, surfactants for organo-modification of layered silicates, and many others [79,80,81,82]. Here, many synthetic and naturally-based cationic polymers, e.g., poly(ethylene imine), poly(amino acids), and chitosan, have been used for aforementioned applications [83,84,85,86,87]. From the family of poly(2-oxazolines), partially hydrolyzed poly(2-alkyl-2-oxazolines) have been used as cationic vectors for gene delivery [88,89,90,91]. From other members of the poly(2-oxazoline) family, PIPOx represents an ideal reactive platform for the preparation of cationic polymers through an addition reaction with various amino acids. Hoogenboom’s group reported the synthesis of cationic amino acid functionalized polymethacrylamide vectors for siRNA transfection [43].

**Figure 14:**
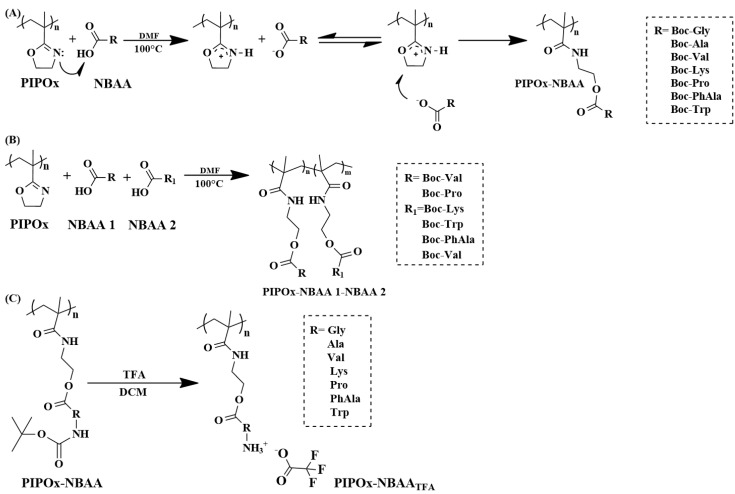
Synthetic pathway for the preparation of cationic polymers based on the addition reaction of PIPOx with selected Boc-protected amino acids. (**A**) the synthesis of fully modified PiPOx homopolymers with N-(tert-Butoxycarbonyl)-amino acids (NBAA), (**B**) the synthesis of fully modified PiPOx copolymers with NBAA, and (**C**) for the preparation of the final cationic polymethacrylamide (co)polymer.

**Figure 15:**
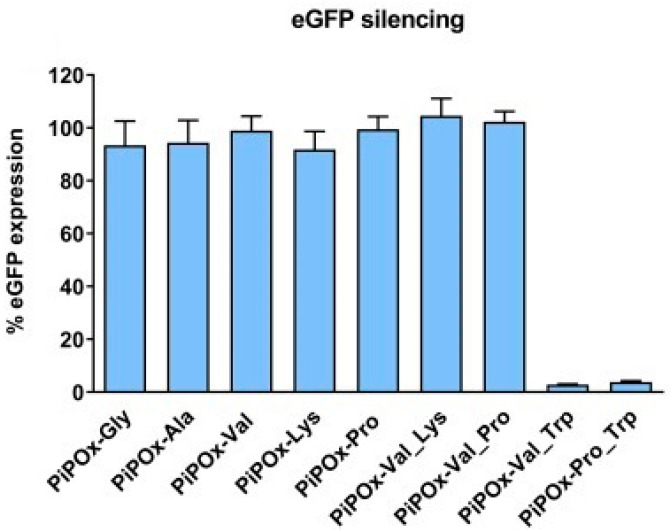
Complexation of PiPOx-NBAA-TFA (co)polymers with siRNA by 1.2% agarose gel electrophoresis. An equal amount of siRNA was mixed at an N/P ratio of 10 with different polymers, as indicated by the names above the wells. Flow cytometric quantification of silencing efficiency in H1299-eGFP cells of polymers that showed acceptable complexation efficiency. Transfection was performed at an siRNA concentration of 50 nM for each sample, and the silencing is expressed as mean ± standard deviation for three technical replicates. Reprinted with permission from Ref. [43]. Copyright 2024 Elsevier.

The prepared cationic copolymers, containing residues of glycine, L-alanine, L-valine, L-serine, L-lysine, L-phenylalanine, and L-tryptophan, were evaluated as non-viral vectors for siRNA transfection (Figure 14). The preliminary experiments with the cationic-charged homopolymers showed that especially the hydrophilic ones can form stable complexes with siRNA, while the hydrophobic amino charged units, such as L-phenylalanine and L-tryptophan, do not complex siRNA (Figure 15). A similar approach was reported for the preparation of cationic polymers based on poly(N-vinylpyrrolidone) (PVP) through an addition reaction with various amino acids [92].

### 5.5. Hydrogels

Hydrogels represent physically or chemically crosslinked three-dimensional polymer networks able to absorb large volumes of water or relevant physiological aqueous fluids, mimicking the extracellular matrix. Hydrogels from synthetic polymers and biopolymers can be useful in many biomedical applications, such as drug delivery, tissue engineering, wound healing, regenerative medicine, cell therapies, and ophthalmology. The majority of studies on synthetic polymer hydrogels are based on the use of PEG [93], PVP [94], poly(vinyl alcohol) (PVA) [95], poly(hydroxyethyl methacrylate) (PHEMA) [96], and, more recently, poly(2-oxazoline)s (POx) [97,98,99,100].

**Figure 16:**
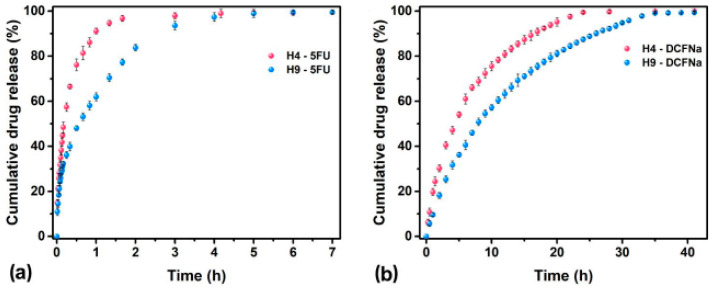
Influence of the dicarboxylic acid chain length on the in vitro release of (**a**) 5-fluorouracyl (5FU) and (**b**) diclofenac sodium over time. Reprinted with permission from Ref. [66]. Copyright 2018 American Chemical Society.

The potential of PIPOx in the development of hydrogels was already shown in the pioneering works of Kagiya, who presented the crosslinking of PIPOx with adipic acid [28]. Covalent crosslinking was performed in N,N-dimethylacetamide at 150 °C, followed by the formation of ester–amide linkages. More recently, Jerca et al. reported the preparation of a hydrogel library using the same crosslinking procedure [66]. Hydrogels were prepared in the crosslinking reaction of PIPOx with eight different non-toxic and bio-based dicarboxylic acids. The equilibrium swelling degree of hydrogels has been modulated by the feed ratio of 2-oxazoline pendant groups and the carboxylic acid groups, as well as chain length in the crosslinker. In vitro studies of the prepared hydrogels demonstrated their non-toxic character and protein-repellent properties. The authors showed that hydrogels prepared by the crosslinking of PIPOx with dodecanedioic acid exhibit the most pronounced thermoresponsive behavior with a volume phase transition temperature of 43 °C, meaning that they could be valuable as ophthalmologic materials or in drug delivery applications. PIPOx hydrogels from succinic and azelaic acids were used to deliver two different hydrophilic model drugs: 5-fluorouracyl and sodium diclofenac. It is obvious that the rate of release was dependent on the chemical composition of hydrogels and also on the type of the drug (Figure 16).

**Figure 17:**
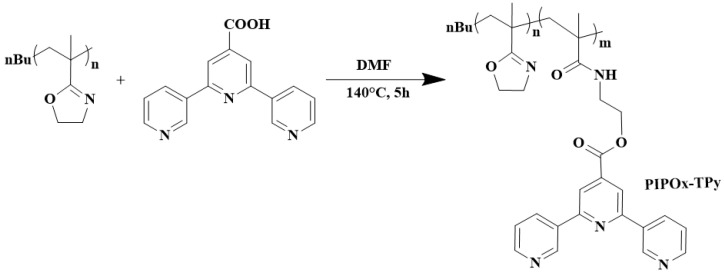
Schematic illustration of the synthesis of PIPOx-functionalized precursors for metal-coordinated supramolecular hydrogels.

Hoogenboom and colleagues reported the development of self-healing hydrogels based on PIPOx [101]. Such materials can be exploited as coatings, actuators for sensors or robotic applications, and wound healing. In the first step, poly(2-isopropenyl-2-oxazoline) was partially modified with 2,2′:6′,2″-terpyridine-4′-carboxylic acid (Figure 17). In the second step, hydrogelation proceeded via the addition of divalent transition metal ions, such as Fe^2+^, Ni^2+^, Co^2+^, and Zn^2+^, resulting in supramolecular hydrogels with tunable rheological properties.

**Figure 18:**
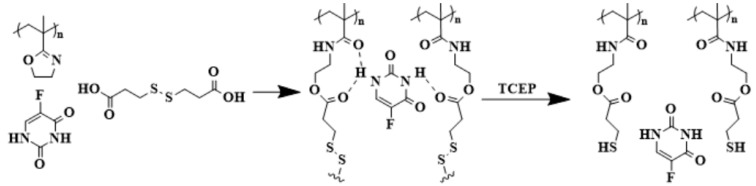
Schematic representation of the preparation of poly(2-isopropenyl-2-oxazoline) molecularly imprinted polymers (PiPOx-MIP) with 5-fluorouracil (5-FU).

The crosslinking reaction of PIPOx with reduction-responsive 3,3′-dithiodipropionic acid has been recently used for molecularly imprinted polymers (MIPs). MIPs were used for the controlled release of 5-fluorouracil (Figure 18) [39]. This concept can also be used for the controlled release of different therapeutic agents or the selective adsorption of hazardous organic molecules from soil or water reservoirs [102,103,104].

**Figure 19:**
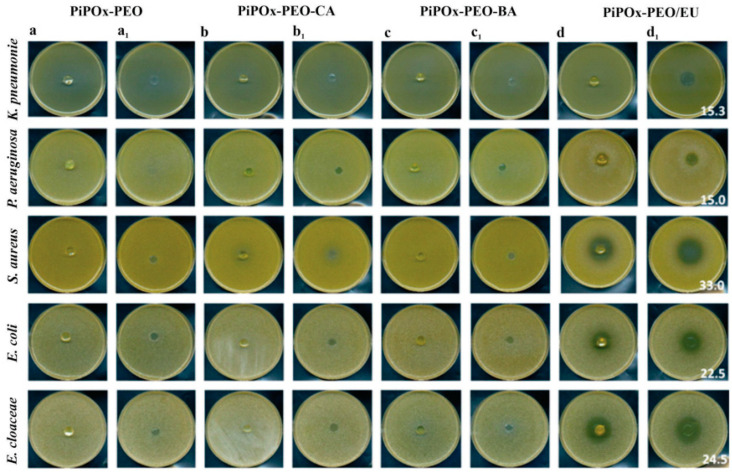
Bacterial growth around the hydrogel discs (**a**–**d**) and under the specimen (**a_1_**–**d_1_**): neat hydrogel (PiPOx–PEO), hydrogel containing bound cinnamic acid (PiPOx–PEO–CA), hydrogel containing bound benzoic acid (PiPOx–PEO–BA), and eugenol-loaded hydrogel (PiPOx–PEO/EU) in the agar diffusion plate test; figures indicate the average diameter of the growth inhibition zone in mm. Reprinted with permission from Ref. [61]. Copyright 2005 Royal Society of Chemistry.

An interesting approach leading to the preparation of tunable properties involves the preparation of segmented networks, where two chemically different polymers are used for the polymer network synthesis. Basko and colleagues prepared segmented polymer networks composed of PiPOx, poly(ethylene oxide) and selected biologically active compounds, such as cinnamic acid, benzoic acid, and eugenol [61]. Polymer networks were prepared by the addition reaction of pendant 2-oxazoline rings of PIPOx with carboxy-terminated poly(ethylene oxide) in DMSO solutions at 140 °C. In the same step, three different carboxyl groups containing biologically active groups, namely, cinnamic acid, eugenol, and benzoic acid, were covalently bound to the PIPOx chains of segmented networks. Their antimicrobial properties were studied against Gram-positive and Gram-negative strains, including *Staphylococcus aureus*, *Pseudomonas aeruginosa*, *Escherichia coli*, *Klebsiella pneumoniae,* and *Enterobacter cloaceae*. All the tested hydrogels showed an antimicrobial effect in the direct contact zone. Moreover, the eugenol-loaded hydrogel expressed a broader bacteriostatic effect, inhibiting microorganism growth beyond the contact zone (Figure 19).

**Figure 20:**
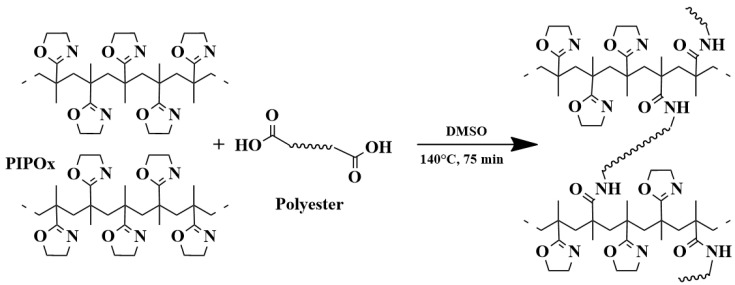
Schematic illustration of the segmented network formation obtained by the addition reaction of PiPOx with dicarboxylic polyesters.

A similar concept was used by the same group for the preparation of biodegradable segmented polymer networks composed of poly(2-isopropenyl-2-oxazoline) and selected aliphatic polyesters [40]. Segmented polymer networks were prepared by crosslinking reactions of PIPOx with carboxy-terminated poly(lactic acid) and poly(caprolactone) (Figure 20). Swelling and mechanical tests, together with in vitro cytotoxicity studies, proved the suitability of these types of materials for biomedical applications, like drug delivery or tissue engineering.

### 5.6. Surface, Molecular, and Bottle-Brush Polymers

Polymer brushes are widely used in biomedical applications to introduce low-protein, non-specific cell adhesion. They are suitable for surface protection of highly sensitive in vitro and in vivo diagnostic devices or other biomedical implantable materials [105,106,107,108]. Poly(ethylene glycol) is one of the most utilized polymers with bioinert and protein-repellent behavior [109,110,111,112,113,114,115]; however, the limitations in the use of PEG as a biomedical polymer are connected with possible oxidative degradation of PEG and the appearance of antibodies against PEGs [116,117,118]. Therefore, poly(2-oxazolines) have been recognized as a promising alternative biomedical grade material.

One of the possible techniques are so-called bottle-brush brushes, consisting of the surface-initiated controlled radical polymerization of 2-isopropenyl-2-oxazoline (IPOx), the alkylation of pendant 2-oxazoline rings, and the subsequent living cationic ring-opening polymerization. Bottle-brush brushes created on SiO_2_ and glassy carbon surfaces were able to effectively prevent biofilm formation by inhibiting protein adsorption on the modified surfaces. Since protein adsorption is considered the initial step in biofilm formation, it is crucial to have surfaces with low protein adsorption to achieve effective anti-fouling properties [65].

Another approach of poly(2-oxazoline)-based bottle-brush brushes reports the synthesis of polished glassy carbon substrates [119]. First, homogeneous and stable PIPOx brush layers with thicknesses of up to 160 nm were created directly onto glassy carbon by the self-initiated photografting and photopolymerization of IPOx. In the second step, the pendant 2-oxazoline rings were used for cationic polymerization of 2-ethyl-2-oxazoline to form a bottlebrush structure attached to glassy carbon.

**Figure 21:**
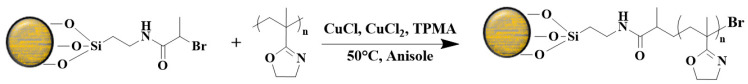
Schematic illustration of the SI-ATRP of IPOx from brominated carbonyl iron particles.

Ilčíková et al. reported the surface-initiated atom transfer radical polymerization (SI-ATRP) of PIPOx onto modified carbonyl-iron (CI) particles to prepare core–shell nanoparticles (Figure 21) [42]. For SI-ATRP, a halogen exchange method using bromide-based initiator-modified carbonyl iron particles has been used. Core–shell particles were received in a controllable manner with two shell thicknesses of 1470 g/mol and 17,000 g/mol, respectively.

The magneto-responsive studies of both neat and CI-PIPOx core–shell particles, investigated as rheological blood-like fluids, showed that the PIPOx shell slightly decreased the yield stresses compared to neat CI-based systems; however, values above 400 Pa could be reached, which is suitable for possible application as an embolization agent.

### 5.7. Polymer Coatings

Polymer coatings represent advantageous methods to protect the surface of materials. In terms of biomedical applications, polymer coatings are, for example, widely used for the immune protection of implantable materials. From a plethora of techniques, cold plasma technologies have been developed for the surface modification of different materials. Plasma polymers of 2-oxazoline monomers (e.g., 2-methyl-2-oxazoline, 2-ethyl-2-oxazoline, or 2-isopropenyl-2-oxazoline) display interesting features that make them good candidates for use in biomedical applications from the point of promoted cell adhesion or reversibly displaying non-biofouling properties [36,120].

**Figure 22:**
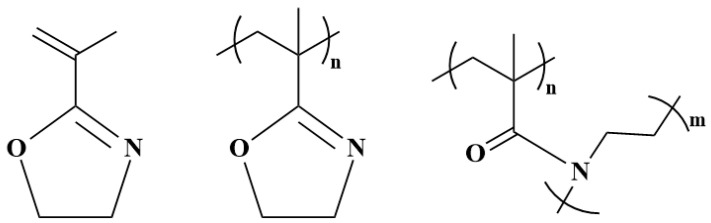
Possible structures of in-plasma polymerized 2-isopropenyl-2-oxazoline.

The plasma polymerization of IPOx proceeds mainly via the polymerization of the double bond, with partial retention of the 2-oxazoline groups (Figure 22). This retention, confirmed by ATR-FTIR and ^1^H NMR spectroscopy, led to the formation of the poly(N-acylethylene imine) structure and depended on the deposition parameters responsible for the monomer fragmentation in the plasma phase. The stability of thin films in physiological conditions was found to be dependent on the fragmentation of the monomer molecules in the plasma phase and on the surface activation of the growing layer during the plasma-on period, which, in turn, depended on the deposition conditions.

## 6. Conclusions and Perspectives

Poly(2-isopropenyl-2-oxazoline) was referred to as a versatile polymer platform for the preparation of advanced materials in various biomedical applications. The possibility to precisely control the polymer molar mass, narrow dispersity, and final architecture through several living/controlled polymerization techniques not sensitive to the presence of a reactive 2-oxazoline ring is advantageous for the preparation of advanced materials. Moreover, PIPOx can be easily modified by addition reactions with carboxylic acids or thiols, providing straightforward access to a wide range of functional polymers. In such a manner, thermosensitive polymers, cationic polymers, drug-conjugates, hydrogels, brushes, and many other architectures can be achieved. PIPOx represents biocompatible and immunocompatible polymers with very good solubility in water and physiological fluids. These features make them ideal candidates for many biomedical applications. This mini-review highlighted possible applications of PIPOx-based materials, such as controlled drug and gene delivery systems, scaffolds for tissue engineering, wound healing materials, actuators for biosensors, and many others.

In spite of many examples of the exploitation of PIPOx in biomedical applications, we believe there is still room for further progress and development on new functionalized materials based on PIPOx chemistry. Moreover, PIPOx can be useful in the future as a platform for the development of new types of smart materials, hybrid materials with inorganics or bio-based materials, nanostructured materials, and many others.

## Data Availability

Not applicable.
